# Agreement between Patient and Proxy Assessments of Quality of Life among Older Adults with Vascular Cognitive Impairment Using the EQ-5D-3L and ICECAP-O

**DOI:** 10.1371/journal.pone.0153878

**Published:** 2016-04-21

**Authors:** Jennifer C. Davis, Ging-Yuek Hsiung, Stirling Bryan, Claudia Jacova, Patrizio Jacova, Michelle Munkacsy, Winnie Cheung, Philip Lee, Teresa Liu-Ambrose

**Affiliations:** 1 Centre for Clinical Epidemiology and Evaluation, University of British Columbia & Vancouver Coastal Health Research Institute (VCHRI), Vancouver, British Columbia, Canada; 2 Djavad Mowafaghian Centre for Brain Health, University of British Columbia, Vancouver, British Columbia, Canada; 3 Aging, Mobility, and Cognitive Neuroscience Lab, University of British Columbia, Vancouver, British Columbia, Canada; 4 Department of Physical Therapy, University of British Columbia, Vancouver, British Columbia, Canada; 5 Centre for Hip Health and Mobility, University of British Columbia, Vancouver, British Columbia, Canada; 6 St Paul's Hospital, Providence Health Care Department of Medicine, Vancouver, Canada; University of Kentucky, UNITED STATES

## Abstract

**Background:**

The assessment of quality of life is critical in ascertaining the benefit of interventions aimed to reduce morbidity among individuals with cognitive impairment. However, the assessment of quality of life is challenging in this population due to the uncertain validity of patient responses as cognitive function declines. Hence, we examined the level of agreement between patient and proxy assessments of health related quality of life (HRQoL) and wellbeing based on the domains that comprise each of these constructs.

**Methods:**

Analysis of baseline data from 71 community-dwelling older adults with mild Vascular Cognitive Impairment (VCI) who participated in a six-month proof-of-concept single-blinded randomized trial. Level of agreement between patient and caregiver ratings of HRQoL (EQ-5D-3L) and wellbeing (ICECAP-O) were compared using raw agreement (%), intraclass correlation coefficient (ICC) and weighted Cohen’s kappa statistic.

**Results:**

Self-care (assessed via the EQ-5D-3L) demonstrated almost perfect raw agreement between the patient and caregiver ratings. Three domains (mobility, pain and anxiety) of the EQ-5D-3L demonstrated fair agreement between the patient and caregiver ratings. Two (attachment and control) of the five ICECAP-O domains demonstrated slight agreement. The ICC indicated good agreement for the EQ-5D-3L and poor agreement for the ICECAP-O.

**Conclusion:**

There is better patient-proxy agreement for the EQ-5D-3L compared with the ICECAP-O among individuals with mild VCI. These findings imply that the ICECAP-O may have limited clinical, research and policy related utility among individuals with mild VCI.

**Trial Registration:**

ClinicalTrials.gov NCT01027858

## Background

Cognitive impairment has substantive effects on healthy ageing–it can negatively impact one’s health related quality of life (HRQoL), and wellbeing. Vascular cognitive impairment (VCI) encompasses all levels of cognitive decline–ranging from mild cognitive deficits to dementia, due to both overt and covert cerebrovascular disease [1]. Dementia, caused by VCI, is the second most common type of dementia worldwide [2–5]. Individuals with cognitive impairment and dementia become dependent on others for their activities of daily living. A common consequence of these changes is decreased HRQoL and wellbeing. In the context of providing care for those with cognitive impairment and dementia, HRQoL and wellbeing are important outcomes to assess and monitor. Notably, HRQoL is identified as an important outcome in interventions aimed at combatting cognitive dysfunction [6]. The gaining recognition of HRQoL and wellbeing as valuable outcome measures places a growing need to compare different modalities of assessing these outcomes (i.e., self-appraisal of HRQoL and wellness across the spectrum of cognitive function (i.e., normal, subjective cognitive decline, mild cognitive impairment, and dementia)). To date, the results of these comparisons are inconclusive, with some studies finding differences in self-appraised HRQoL between individuals of different cognitive status, while others have found no differences [6–9]. A contributing factor to the variable findings may be inaccurate self-appraisal of HRQoL due to cognitive decline. Practically, enlisting the help of a caregiver or proxy to respond on behalf of the patient may be a solution to help avoid systematic exclusion of participants with cognitive impairment; however, it is first critical to ascertain the level of agreement prior to providing recommendations for HRQoL and wellbeing assessment.

Impaired cognition may impede an individual’s ability to accurately self-assess their HRQoL and wellbeing. This poses a major challenge facing economic evaluations of interventions targeting cognitive impairment and dementia prevention [10,11]. Hence, it is important to gain better understanding of measures that provide valid and reliable assessments of HRQoL and wellbeing among this population. Wellbeing reflects the meaning an individual attaches to the effects of a condition or disease on themselves. Wellbeing is distinct from HRQoL because wellbeing is not focused on health status alone. Many health care issues among older adults (i.e., cognitive decline, falls) [12] are accompanied by forms of care such as nursing homes, residential care, family member caregiving thus combining both health and social care (i.e., wellbeing and quality of life are used interchangeably here). Wellbeing has gained increased importance as an outcome in intervention studies, given that many interventions have impact extending beyond an individuals health alone [13–16]. Wellbeing is a construct that takes a broader approach than considering health alone. One method of assessment of an individual’s wellbeing focuses on a capability approach. Specifically, capabilities reflect an individual’s ability to do a specific task; whereas, a functioning reflects whether or not the individual does a specific task or is in a specific state. Sen [17] emphasizes that an individual’s capabilities are most useful in assessing and comparing impact of interventions. Wellbeing can be measured using the ICECAP-O, an index of capability. The ICECAP-O is a preference-based outcome measure designed to provide a broader assessment of an individual’s quality of life or wellbeing [18,19]. This instrument is conceptually linked to Sen's capability approach, which defines wellbeing in terms of what individuals are able to do (i.e., capabilities), not what individuals actually do (i.e., functionings).

Health related quality of life is a construct that represents an individual’s quality of life related to health factors alone and it is an important issue in the context of dementia care. It is commonly assessed using the EQ-5D three level version (3L). The EQ-5D three level (3L) version is a preference based utility instrument that captures 243 health states [20] to ascertain an individual’s HRQoL according to five domains: mobility, self-care, usual activities, pain and, anxiety or depression. The EQ-5D is one of the most widely used generic instrument that uses a utility-based scoring approach, yielding a single summary score (i.e., health-state utility value) on a common scale to facilitate comparison across different health conditions and patient populations [20].

Impaired cognition may impede an individual’s ability to accurately self-assess their HRQoL and wellbeing. Assessment of wellbeing and HRQoL is often neglected in studies among individuals with mild cognitive impairment [21,22]. Mild cognitive impairment is an intermediate stage between normal cognitive decline associated with aging and more serious cognitive decline associated with dementia. Symptoms may include problems with memory, language, thinking and judgment that are beyond normal aging related decline. Importantly, individuals with mild cognitive impairment may actually represent an ideal target population both for interventions and for potentially valid and reliable HRQoL and wellbeing assessment given that they have not yet crossed the dementia threshold. There is increasing emphasis on wellbeing more broadly, compared with HRQoL alone, as a critical outcome measure for economic evaluations and among specific populations such as older adults with mild cognitive impairment [23]. However, before this emphasis is promoted among individuals with cognitive impairment understanding the agreement of patient and proxies is critical. Given that the ICECAP-O is a relatively new instrument, the literature is relatively devoid of patient versus proxy comparisons [24,25]. Further, interventions aimed at combatting cognitive decline are expected to result in gains or losses beyond HRQoL alone; therefore, we assessed wellbeing more broadly using the ICECAP-O. Thus, the objective of our study was to determine the baseline level of agreement between patient and proxy assessments of HRQoL (assessed using the EQ-5D-3L) and wellbeing (assessed using the ICECAP-O).

## Methods

### Study Design

The analyses presented in this paper are cross-sectional comparisons of 71 older adults enrolled in a six-month proof-of-concept single-blinded randomized trial of exercise. All participants had mild cognitive impairment primarily due to vascular burden in the brain (i.e., mild vascular cognitive impairment)[26].

### Inclusion/Exclusion Criteria

The inclusion/exclusion criteria were previously defined in the published study protocol [26]. Individuals were considered eligible if they fulfilled the diagnostic criteria for VCI which requires the presence of both cognitive syndrome and small vessel ischaemic disease [27]. Clinical diagnosis of VCI was made by neurologists and geriatricians based on the presence of presence of both small vessel ischemic disease and cognitive syndrome. Small vessel ischemic disease was defined as evidence of relevant cerebrovascular disease by brain computed tomography (CT) or magnetic resonance imaging (MRI) defined as the presence of both: 1) Periventricular and deep white matter lesion (WML): patchy areas of low attenuation or diffuse symmetrical areas of low attenuation with ill defined margins extending to the centrum semiovale, plus at least one lacunar infarct; 2) Absence of cortical and or cortio-subcortical non-lacunar territorial infarcts and watershed infarcts, hemorrhages indicating large vessel disease, signs of normal pressure hydrocephalus, or other specific causes of WML (i.e. multiple sclerosis, leukodystrophies, sarcoidosis, brain irradiation). Cognitive syndrome was defined as Montreal Cognitive Assessment (MoCA)[28] score less than 26/30 at baseline. Progressive cognitive decline was confirmed through medical records or caregiver/family member interviews.

Individuals needed to have a caregiver, family member, or friend who interacted with him/her on a weekly basis. The caregivers had to be able to read, write, and speak English in which the questionnaires were provided with acceptable visual and auditory acuity. Caregivers completed the questionnaires from their own perspective of the participant (i.e., proxy’s perspective).

Ethical approval has been obtained from the Vancouver Coastal Health Research Institute (V07-01160) and the University of British Columbia’s Clinical Research Ethics Board (H07-01160). All participants and/or their proxies (i.e., caregiver/family member) signed written and informed consent prior to their participation in this study.

### Measurements

Our analyses used data acquired at baseline of this randomized controlled trial. Baseline measurements were obtained prior to randomization. Assessors were trained and standardized protocols were used.

### Primary Outcome Measures

#### Health Related Quality of Life and Wellbeing

The primary outcome variables of interest for this cross-sectional analysis were the EQ-5D-3L and the ICECAP-O. Patients and proxies (i.e., caregivers) completed the EQ-5D-3L first and subsequently the ICECAP-O using paper versions that were given to them at their initial baseline assessment. The proxies were read a script instructing them to provide their own perspective (i.e., proxy’s perspective) of the patients’ abilities or functioning. The participant and the caregiver were asked not to discuss their questionnaire response items with each other during completion of the questionnaires. Trained research assistants moderated all questionnaire completion.

#### EQ-5D-3L

We assessed HRQoL using the EQ-5D three level version (3L). The EQ-5D-3L is a short five item multiple choice questionnaire that measures an individual’s HRQoL and health status according to the following five domains: mobility, self-care, usual activities, pain and anxiety/depression [29]. Each domain has three possible response options indicating no problems, some problems or severe problems. The EQ-5D-3L health state utility values (HSUVs) are bounded by a range from -0.54 to 1.00. A score of less than zero is indicative of a health state worse than death. The HSUVs represent values that individuals within society assign. There are UK[29] and Canadian[30] societal valuations for given health states.

#### ICECAP-O

We assessed quality of life and wellbeing using the ICECAP-O [18,31,32]. The ICECAP-O value system defines 1024 unique states valued using a best-worst scaling valuation method among older adults in England [18]. The value system provides a single summary index score, anchored at zero (‘no capability’) and 1.0 (‘full capability’). The ICECAP-O covers attributes of capability found to be important determinants of quality of life more broadly among older adults in the UK [18,32]. It includes the following five attributes: 1) attachment (love and friendship), 2) security (thinking about the future without concern), 3) role (doing things that make you feel valued), 4) enjoyment (enjoyment and pleasure) and 5) control (independence). The ICECAP-O is a five item multiple choice questionnaire where each attribute has four possible response options.

### Descriptive Variables

General health and socioeconomic status were ascertained by a questionnaire. Participants received a clinical assessment with a neurologist or geriatrician (G-YRH and PL) at baseline to confirm current health status and eligibility for study, including clinical impressions of overall cognitive and functional status [33]. Physical function was assessed using the Physiological Profile Assessment (PPA) [34] (Prince of Wales Medical Research Institute, Randwick, Sydney, NSW, Australia) to assess for physiological falls risk. The PPA is a valid and reliable measure of falls risk [35,36]. Based on a participant’s performance in five physiological domains–postural sway, reaction time, strength, proprioception, and vision–the PPA computes a falls risk score (standardized score) that has a 75% predictive accuracy for falls among older people [37,38]. A PPA Z-score ≥ 0.60 indicates high physiological falls risk [39].

#### Cognitive Function

Global cognitive function were assessed using the MMSE [40] and the MoCA [41]. The Alzheimer Disease Assessment Scale (ADAS-Cog) was the primary cognitive outcome of the RCT [42]. The ADAS-Cog assesses memory, language, and praxis with 11 items: word recall task, naming objects and fingers, following commands, constructional praxis, ideational praxis, orientation, word recognition task, remembering test directions, spoken language, and comprehension. Scores range from 0 to 70, with higher scores indicating greater severity of cognitive impairment [42]. The ADAS-Cog has marked advantages, including its acceptable reliability and validity, sensitivity to longitudinal changes in cognitive performance, and responsiveness to treatment effects [43]. The ADAS-Cog has been a significant outcome measure in numerous trials in AD [44,45], vascular dementia [46,47], SIVCI [48], and MCI [49].

### Statistical Analyses

All statistical analyses were performed using STATA version 11.0. For data that were normally distributed we report mean and standard deviation and frequencies (%) depending on the measure. For data that were non-normally distributed we report median and interquartile range. Agreement between the patient and the proxy on each item of the EQ-5D-3L and the ICECAP-O was assessed using the weighted kappa. The levels of agreement for the weighted Kappa were based on the following criteria: < 0 no agreement; 0–0.20 slight; 0.21–0.40 fair; 0.41–0.60 moderate; 0.61–0.80 substantial and; 0.81–1 almost perfect agreement. For the EQ-5D-3L, both the Canada and UK valuations were tabulated for the global scores. For the ICECAP-O, UK valuations were tabulated because Canadian valuations are not yet available. A p-value of 0.05 was deemed statistically significant. Based on the global scores for the EQ-5D-3L and the ICECAP-O, we also calculated the intraclass correlation coefficients (ICC). ICC values less than .40 indicates poor agreement, between .40 and .59 indicated fair agreement, between .60 and .74 indicated good agreement, and between .75 and 1.0 indicates excellent agreement.

## Results

### Participants

Table 1 reports descriptive statistics for participants at baseline. At baseline, participants self-reported their HRQoL at 0.805 (SD: 0.100) and their caregiver reported their HRQoL at 0.810 (SD: 0.100). At baseline, participants self-reported their wellbeing at 0.848 (SD: 0.110) and their caregiver reported the participants wellbeing at 0.854 (SD: 0.090). Further, the mean MoCA score was 21 (SD: 4) and the mean MMSE was 26 (SD: 3). Table 2 reports the individual domain frequencies (%) of the EQ-5D-3L and the ICECAP-O for patients and their proxies as well as the raw agreement (%) between patients and their proxies. Of note, we were unable to establish contact with 6 proxies. For the EQ-5D-3L, almost perfect raw agreement was observed for the self-care domain (96%) agreement and high raw agreement (%) was observed for the mobility domain (76%). The raw agreement (%) was equal or greater to 65% for all domains of the EQ-5D-3L. For the ICECAP-O, the highest raw agreement (%) for the ICECAP-O was observed for the control (52%) and attachment (45%) domains. The remaining domains (security, role, enjoyment) of the ICECAP-O demonstrated poor raw agreement between 32–39%.

**Table 1:** Characteristics of the VCI participants at baseline.

Variable at Baseline	Frequency (%) or Mean (SD) or Median (IQR)
Caregiver classification	
*Family- child*	17 (31%)
*Family-spouse*	24 (44%)
*Family-other*	3 (5%)
*Friend*	9 (16%)
*Employee*	2 (4%)
EQ-5D-3L* (participant)	0.805 (0.100), 0.826 (0.100)
EQ-5D-3L* (caregiver)	0.810 (0.100), 0.835 (0.108)
ICECAP-O (participant)	0.848 (0.110), 0.868 (0.080)
ICECAP-O (caregiver)	0.854 (0.090), 0.853 (0.700)
Age (years)	74 (8)
Function Comorbidity Index	3.7 (1.6), 4(2)
MMSE (max 30 pts)	26 (3), 27 (4)
MoCA	21 (4), 22 (5)
ADAS-Cog	11 (5), 10(6)
6-Minute Walk	494 (97)
PPA	1.0 (1.5)

* UK Valuations

MMSE: Mini Mental State Examination

MoCA: Montreal Cognitive Assessment

ADAS-Cog: Alzheimer Disease Assessment Scale

PPA: Physiological Profile Assessment

**Table 2:** Distributions of Individual Domains of the EQ-5D-3L and the ICECAP-O.

Variable at Baseline	Participant Frequency(%)	Caregiver Frequency(%)	Raw Agreement Frequency(%)
EQ-5D-3L* **Global Score**			
**Mobility**	n = 70	n = 64	n = 62, 47 (76%)
I have no problems in walking about	53 (75%)	49 (77%)	
I have some problems in walking about	17 (%)	15 (23%)	
I am unable to walk about	0 (0%)	0 (0%)	
**Self-care**	n = 70	n = 64	n = 62, 58 (96%)
I have no problems washing or dressing myself	68 (97%)	52 (81%)	
I have some problems washing or dressing myself	2 (3%)	12 (19%)	
I am unable to wash or dress myself	0 (0%)	0 (0%)	
**Usual activities**	n = 70	n = 64	n = 62, 40 (65%)
I have no problems doing my usual activities	50 (71%)	62 (97%)	
I have some problems doing my usual activities	20 (29%)	2 (3%)	
I am unable to do my usual activities	0 (0%)	0 0 (0%)	
**Pain**	n = 70	n = 64	n = 62, 41 (66%)
I have no pain or discomfort	38 (54%)	52 (81%)	
I have moderate pain or discomfort	30 (43%)	12 (19%)	
I have extreme pain or discomfort	2 (3%)	0 0 (0%)	
**Anxiety/Depression**	n = 70	n = 64	n = 62, 42 (68%)
I am not anxious or depressed	49 (70%)	44 (69%)	
I am moderately anxious or depressed	20 (29%)	18 (28%)	
I am extremely anxious or depressed	1 (1%)	2 (3%)	
ICECAP-O*			
**Attachment**	n = 70	n = 64	n = 62, 28 (45%)
I can have all of the love and friendship that I want	27 (39%)	27 (42%)	
I can have a lot of the love and friendship that I want	36 (51%)	31 (48%)	
I can have a little of the love and friendship that I want	5 (7%)	6 (9%)	
I cannot have any of the love and friendship that I want	2 (3%)	0 (0%)	
**Security**	n = 70	n = 64	n = 62, 22 (35%)
I can think about the future without any concern	11 (16%)	9 (14%)	
I can think about the future with only a little concern	29 (41%)	22 (34%)	
I can think about the future with some concern	25 (36%)	21 (33%)	
I can think about the future with a lot of concern	5 (7%)	12 (19%)	
**Role**	n = 70	n = 64	n = 62, 20 (32%)
I am able to do all of the things that make me feel valued	21 (30%)	19 (30%)	
I am able to do many of the things that make me feel valued	37 (53%)	35 (55%)	
I am able to do a few of the things that make me feel valued	11 (16%)	10 (16%)	
I am unable to do any of the things that make me feel valued	1 (1%)	0 (0%)	
**Enjoyment**	n = 70	n = 64	n = 62, 24 (39%)
I can have all of the enjoyment and pleasure that I want	13 (19%)	19 (30%)	
I can have a lot of the enjoyment and pleasure that I want	44 (63%)	40 (63%)	
I can have a little of the enjoyment and pleasure that I want	13 19%)	5 (8%)	
I cannot have any of the enjoyment and pleasure that I want	0 (0%)	0 (0%)	
**Control**	n = 70	n = 64	n = 62, 32 (52%)
I am able to be completely independent	28 (40%)	31 (48%)	
I am able to be independent in many things	40 (57%)	29 (45%)	
I am able to be independent in a few things	1 (1%)	3 (5%)	
I am unable to be at all independent	0 (0%)	1 (2%)	

* UK Valuations

### Patient and Proxy Agreement for the EQ-5D-3L

**Table 3** reports the kappa statistic comparing the participant rating with the caregiver rating for each domain of the EQ-5D-3L. Three domains (mobility, pain and anxiety) within the EQ-5D-3L demonstrating fair agreement and usual activities demonstrating slight agreement between the patient and caregiver ratings. Of note, the self-care domain demonstrated “no agreement” in contrast to the raw agreement of 96% from Table 2 indicating a kappa paradox phenomenon. The ICC for the global health state utility score for the EQ-5D was 0.60 (95%CI: 0.41–0.74) indicating good agreement on an individual basis.

**Table 3:** Kappa.

Instrument	Kappa	P-value	Level of Agreement**
**EQ-5D-3L**			
Mobility	0.355*	0.0026	Fair agreement
Self-care	-0.033	0.6035	No agreement
Usual activities	0.0541	0.3251	Slight agreement
Pain	0.348*	0.0017	Fair agreement
Anxiety/Depression	0.276*	0.0092	Fair agremment
**ICECAP-O**			
Attachment	0.0509	0.3077	Slight agreement
Security	0.0783	0.1574	No agreement
Role	-0.1281	0.9121	No agreement
Enjoyment	-0.1487	0.9444	No agreement
Control	0.1062	0.1708	Slight agreement

* p<0.05

** Level of agreement

< 0 no agreement

0–0.20 slight

0.21–0.40 fair

0.41–0.60 moderate

0.61–0.80 substantial

0.81–1 almost perfect agreement.

### Patient and Proxy Agreement for the ICECAP-O

Three (security, role and enjoyment) of the ICECAP-O domains demonstrated no agreement between patient and caregiver ratings (Table 3). Two domains (attachment and control) demonstrated slight agreement between the patient and caregiver ratings. The findings concur with the raw agreement (%) observed in Table 2. The global ICECAP-O score did not demonstrate significant agreement between the patients and the caregivers ratings. The ICC for the global score for the ICECAP-O was 0.29 (95%CI: 0.05–0.50) indicating poor agreement on an individual basis.

## Discussion

### Principal findings

In this study, we provide novel evidence that the level of agreement between patient and proxy for the EQ-5D-3L was significantly better than the level of agreement observed for the ICECAP-O. The results based on individual domain analysis and global score analysis were consistent. The level of raw agreement between patient and proxy for the EQ-5D-3L demonstrated almost perfect agreement for the self-care domain and high agreement for the mobility domain. Specifically, for the EQ-5D-3L, patient-proxy agreement assessed using the kappa statistic was primarily driven by three domains (mobility, pain and depression/anxiety). In contrast, there was minimal patient-proxy agreement for the ICECAP-O with two domains (attachment and control) demonstrating slight agreement. Ultimately, these preliminary data suggest the EQ-5D provides stronger agreement between patient and proxies than the ICECAP-O.

This study has the following limitations. In this study we report UK[29] values given that there are no Canadian valuations for the ICECAP-O. Second, there is a discrepancy in the self-care domain findings between the raw agreement and the kappa statistic indicating a kappa paradox phenomenon [50]. This is not surprising given that the distributions that comprise the EQ-5D were highly skewed creating a symmetrical imbalance [50]. As such, the kappa statistic for self-care should be interpreted with caution. Further, the number of levels for each domain of the EQ-5D and ICECAP-O is an important consideration. Specifically, agreement is generally higher when more levels are included which would give the ICECAP-O a better 'chance' at greater agreement. The sample size, although appropriate for the primary outcome measure for the purpose of the randomized study, may have limited power to detect a statistically significant difference. Therefore, finding or not finding significant evidence in support of agreement between the EQ-5D and the ICECAP-O may limit the generalizability of the study findings. Specifically, our findings only apply to the population of individuals’ with mild VCI. Also relating to sample size, is the issue of type of caregiver classification (i.e., family, friend, employee) and subgroup analyses to understand whether level of agreement differs based on type of caregiver. It may be possible that the level of agreement may differ depending on the type of caregiver.

### Comparison with other research

Previous research, limited in scope, has demonstrated that substantial differences exist between patient and proxy ratings for the EQ-5D and the ICECAP-O among individuals with dementia [7,24]. Specifically, for the EQ-5D-3L, data provided by a clinician proxy had higher construct validity for observable dimensions (i.e., mobility, self-care) while data provided by a family proxy had higher construct validity for less observable dimensions (i.e., usual activities, anxiety/depression) [7]. For the ICECAP, individuals with dementia found that proxy responses were driven by gender and experience of the proxy [24]. Importantly, much comparative research has focused on dementia and ignored populations, which may represent a critical interface–individuals with MCI such as VCI. Our findings suggest that the domains of mobility, pain and anxiety/depression drive patient-proxy agreement for the EQ-5D. The lack of any patient-proxy agreement for the ICECAP-O may have important implications for interpreting the findings in policy and practice.

Of note, previous research has not identified minimal clinically important differences (MCID) for the ICECAP-O. A MCID for the EQ-5D-3L of 0.03 has been reported as clinically important. Given that the scales are different for the ICECAP-O and the EQ-5D-3L, we cannot directly compare these differences. However, it is possible that although the global scores between the patient and proxy for the EQ-5D-3L and the ICECAP-O are very similar, this still could equate to low levels of agreement. Future work exploring the MCIDs of each of these populations among individuals with VCI would assist in interpreting these results further. It may be that when considering the global scores for the EQ-5D-3L and the ICECAP-O, that domain or attribute specific biases are minimized. From a policy perspective, given that treatment comparisons are made at a group level, it will be essential to understand the validity and reliability of both of these instruments.

### Implications for policy and practice

Individuals perceptions of their ability to function may be more quickly affected compared with their concrete ability to a more directly observable change such as “I have no problems walking about” (EQ-5D-3L). Given that the ICECAP-O taps into individuals capability to achieve “functionings” rather than their concrete ability to perform specific functions may explain the lack of significant agreement between patients and proxies in this group. Briefly, it is conceivable that individuals found completing the ICECAP-O a more difficult cognitive task as compared to the EQ-5D-3L. Our previous research examining the comparative feasibility of these two instruments and they item completion rates support this hypothesis [51]. Specifically, the domains such as “attachment”, security, and “role” may be rather abstract concepts to patients with VCI because patients with VCI have frontal deficits resulting in loss of ability to understand and critically appraise themselves on these domains (i.e., patients no longer have an accurate self-assessment of their wellbeing). Further, it could be difficult for proxies to observe and judge. For example, it is conceivable that caregivers in general are almost certainly less able to judge such internal states such as how often a patient is thinking about the future without concern or doing things that make him or her feel valued, as compared to more objective, external measures such as a patient's mobility and abilities to perform self-care. Hence, these data provide a cautionary tale highlighting that the ICECAP-O may not be a suitable tool in a clinical, research or health policy setting among patients with VCI.

### Unanswered questions and future research

Due to the frontal deficits in patients with VCI, they may lose their ability to assess their wellbeing sooner than their ability to assess HRQoL. It is because of this reason that proxy assessment is deemed useful (ie., patients with (mild) dementia cannot be relied upon to give an accurate and unbiased assessment of their own wellbeing). Yet, proxies also cannot give accurate and unbiased estimates of the patient’s wellbeing or HRQol. Hence, the questions remains: which of these biases is stronger? The answer to this question depends on the patient population. It may be possible that the two biases for patient and proxy for the ICECAP-O are equal. Ultimately, a choice needs to be made as to whether the patient or the proxy responses are less likely to be biased based on the instrument and the population. The next essential step is to examine patient-proxy agreement along different levels of the dementia spectrum to determine the threshold of cognitive impairment at which patient responses are more biased than proxy responses. Future work should examine how agreement differs between subgroups with higher cognitive deficits compared with subgroups with lower cognitive deficits. Longitudinal measurement of differences between patient and proxies will help inform these thresholds. In addition, future research is needed to explore and classify different types of proxies and their level of agreement with each instrument as these are likely different.

We acknowledge the prevailing concern that the ADAS-Cog may lack sensitivity to mild changes in cognitive function and that the Vascular Dementia Assessment Scale cognitive subscale (VADAS-Cog) may be more sensitive endpoint for individuals with vascular burden of the brain [43]. However, at the time of initiating this study, VADAS-Cog data from longitudinal and intervention studies were lacking.

## Conclusion

This study demonstrates better patient-proxy agreement for the EQ-5D-3L compared with the ICECAP-O among individuals with VCI. According to the raw agreement (%) patient-proxy agreement for the EQ-5D-3L appears to be primarily driven by two domains (mobility and self-care). From a clinical perspective, it is important to identify which domains (i.e., mobility and self-care) have the greatest level of agreement. However, evidence based on the kappa statistic indicates that pain and anxiety/depression may also play an important role. On the contrary, there is minimal patient-proxy agreement based on the raw agreement (%) and kappa statistic with the ICECAP-O, which may have important implications for the use of the ICECAP-O in this population. In summary, given the good level of agreement, the EQ-5D-3L may be a suitable instrument for completion by a proxy among individuals with mild VCI. Of note, the findings of this study may not be generalizable to broader populations of dementia.
